# A Deep Cryptographic Framework for Securing the Healthcare Network from Penetration

**DOI:** 10.3390/s24217089

**Published:** 2024-11-04

**Authors:** Arjun Singh, Vijay Shankar Sharma, Shakila Basheer, Chiranji Lal Chowdhary

**Affiliations:** 1 Department of Computer and Communication Engineering, Manipal University Jaipur, Jaipur 303007, India; arjun.singh@jaipur.manipal.edu; 2 Department of Information Systems, College of Computer and Information Science, Princess Nourah bint Abdulrahman University, Riyadh 11671, Saudi Arabia; sbbasheer@pnu.edu.sa; 3 School of Computer Science Engineering and Information Systems, Vellore Institute of Technology, Vellore 632014, India

**Keywords:** cryptography, healthcare, twofish security, hash function

## Abstract

Ensuring the security of picture data on a network presents considerable difficulties because of the requirement for conventional embedding systems, which ultimately leads to subpar performance. It poses a risk of unauthorized data acquisition and misuse. Moreover, the previous image security-based techniques faced several challenges, including high execution times. As a result, a novel framework called Graph Convolutional-Based Twofish Security (GCbTS) was introduced to secure the images used in healthcare. The medical data are gathered from the Kaggle site and included in the proposed architecture. Preprocessing is performed on the data inserted to remove noise, and the hash 1 value is computed. Using the generated key, these separated images are put through the encryption process to encrypt what they contain. Additionally, to verify the user’s identity, the encrypted data calculates the hash 2 values contrasted alongside the hash 1 value. Following completion of the verification procedure, the data are restored to their original condition and made accessible to authorized individuals by decrypting them with the collective key. Additionally, to determine the effectiveness, the calculated results of the suggested model are connected to the operational copy, which depends on picture privacy.

## 1. Introduction

Intending to identify imperfections, vulnerability assessment is a proactive method of locating and taking advantage of weaknesses in information assets from an attacker’s viewpoint [1]. Penetration testing is essential to ensure the reliability, accessibility, and traceability (IACR) of information security in today’s digital environment [2]. This is particularly accurate given that the European General Data Protection Regulation has become legally binding for companies and associations. A wide range of vulnerability testing alternatives are available [3]. Platforms such as Kali Linux, equipped with security tools like Nmap, offer specialized tools for conducting attacks. The penetration testing process identifies and evaluates system vulnerabilities and determines suitable remedial actions [4]. They imitate several kinds of system assaults. Through these procedures, the examiner can systematically and carefully identify the vulnerabilities. These produce information on vulnerabilities in the systems that need to be fixed so that management is aware of the problems [5]. This serves as a risk assessment for verifying network security. Organizations find penetration testing crucial, yet the process and resources are expensive [6]. Thus, a specialized penetration testing technique is required to safeguard devices and systems and guarantee network and information security quickly and affordably [7]. Internet usage is becoming more and more common. For this reason, data security is crucial to thwarting cybercriminals’ attempts [8]. Specialists perform penetration tests to identify and address network vulnerabilities before criminals attempt to exploit them [9].

A network may be a LAN, WLAN, WAN, or Internet of Things network. An internet penetration examination was recommended as an ethical measure to assess the potential hazards in the event of unauthorized entry into the company’s technological infrastructure or network [10]. Furthermore, this authorized cyber-attack simulation helps develop a strategy to fix security flaws in the IT infrastructure before the real assault occurs. The so-called ethical hackers, skilled security professionals, handle it [11]. Protecting data and ensuring general security are the goals of network penetration testing, particularly when it comes to handling sensitive data. Traditional viruses or malware, improperly set fire-walls, and SQL injections are a few examples [12]. To guarantee long-term security, certain policies also require ongoing maintenance and network penetration testing [13].

Network security penetration technologies research focuses on data processing, approach and method development, and modeling network attack detection [14]. In particular, data mining technology is crucial to network attack detection techniques [15]. Vulnerability detection technology finds flaws in host systems or remote hosts, creates repair plans, and guarantees the security of computer systems [16]. It helps generate security standards to prevent network harm and gives staff members in charge of network security management in-depth knowledge about system vulnerabilities [17]. Nevertheless, current vulnerability detection algorithms frequently identify group vulnerabilities without determining their connection [18]. Conventional vulnerability detection methods are limited to identifying known vulnerabilities, and simplistic vulnerability risk assessments fail to accurately depict the network’s overall security status [19]. Conventional network security technology cannot effectively protect intricate network architectures [20]. The Health Insurance Portability and Accountability Act of 1996 (HIPAA) safeguards medical coverage for employees and their families who experience employment changes or loss of medical coverage. It restricts new health plans from denying coverage based on pre-existing conditions. It also mitigates healthcare fraud and abuse, reforms medical liability, and simplifies administration by mandating the establishment of national standards for internet medical services and national identities for providers, hiring managers, and health insurers. It establishes regulations for pre-tax healthcare expenses accounts. It implements modifications to health insurance legislation and adjustments for medical insurance deductions. It provides regulations for collective health insurance schemes that offer adjustments for health insurance. It regulates coverage for life insurance policies owned by the company. It establishes regulations for the treatment of individuals lacking legitimacy in the United States and rescinds financial institution regulations pertaining to interest apportionment.

A technique for assessing network security associations is suggested to tackle this issue. In previous research works, the crypto models were merged with the mathematical model to achieve the finest outcome. However, in some cases, decryption is not possible for those instances. Here, the key novelty of the work is designing the intelligent cryptosystem GCbTS to secure medical image data. Before this research study, the graph convolutional system with twofish was not implemented in any study, so it is the prime novelty of the system. This approach is more accurate in reflecting network security status and is based on conventional vulnerability detection. The following is a summary of this work’s contribution:

- Initially, medical image data were evaluated and trained according to the Python system;
- Consequently, a novel GCbTS is designed with the required crypto-security constraints;
- Henceforth, the hash 1 is found, and the data are encrypted and kept in an unreadable format;
- A hash 2 calculation is done to decrypt the data, verifying whether hash 1 is equal to hash 2;
- If hash 1 and hash 2 are equal, the secret key is shared for decryption;
- Finally, metrics like encryption and decryption time, PSNR, MSE, error rate, and computation time are compared with other models.

## 2. Related Work

To apply reinforcement learning (RL) techniques to the field of cyber security, Ghanem et al. [21] presented an innovative strategy. Using a hierarchical representation of the complex POMDP domain is the best way to solve large Partially Observable Markov Decision Process (POMDP) problems when scaling up is a problem. By separating the system into security clusters, IAPTF gains the ability to manage every one of them independently. This comprehensive approach covers all potential basic and advanced attack techniques, guaranteeing a success rate that matches or surpasses that of a professional moral hacker. The IAPTF is a flexible and all-encompassing framework that reveals complex situations and frees human specialists from monotonous jobs. The proposed technique’s inherent drawback is its limited coverage of attack vectors, which could lead to the absence of certain advanced attack vectors that a human hacker could exploit.

Preetha et al. [22] suggested a blockchain-enabled smart contract security framework for medical applications, reducing the decryption overhead and preventing unwanted access to medical records through data exchange. To improve decryption performance, the system uses a validation contract to assess participant rights and a decryption contract for partial decryption. The system performs better than conventional systems regarding file loss rate, latency, and security. The solution lowers the cost of scheduling and transmitting facsimiles, improving network performance. However, the plan still has issues with network enactment and security enactment. According to experimental data, the approach can offer safe and dependable medical record exchange with fine-grained access control. Before widespread deployment, there are still important issues to resolve, such as human factors that could limit digital platform usage.

Alabbad et al. [23] provided a brand-new penetration testing framework that draws inspiration from software engineering’s parallel testing methodology. The framework takes a target network’s collection of resources and policies and applies an algorithm to create a secure network. The network is evaluated based on its security procedures, segmentation strength, and compliance with the DD strategy compared to a robust network of a similar nature. The approach suits dynamic networks due to its automation capabilities. Ensuring the security of the transferred data between identical twins and target networks is of utmost importance. Two key concerns are the reliability of the permissions policies and the trustworthiness of the risk evaluation suggestions for improvement.

Specifically, Hidayanto et al. [24] introduced a computerized mapping tool that enhances the efficiency of the penetration testing process, especially for complex websites with numerous functionalities. A technique used by the OWASP Juice Shop and Startrek Payroll requires only 0.21 s to find individual test cases and 0.07 s to transfer specific endpoints. In contrast, manually converting requires 12 min and 1.79 s. Level customization improves the process time by mapping fewer test cases than the original level. The JSON-stored mapping pattern facilitates the modification of vulnerability patterns and adds test cases from other standards for penetration testers. This program also makes it easy to record test results. With 100% accuracy, automatic mapping [24] finds every potential test case for every endpoint more quickly than manual mapping.

According to a study by Pandey et al. [25], implementing blockchain technology can effectively detect and prevent the distribution of counterfeit medications within medical networks. The solution’s highly decentralized network has 11 machine nodes that link together. It is extremely secure, even with its demanding computational requirements. Intelligent mobile applications can access the technology for QR code scanning, but they cannot prevent using fake or illegal medications before purchase. However, society can contribute to the system by accepting blockchain-authorized payments. Other industries, such as election management and courier cargo tracing, could benefit from blockchain’s resilience and ledger-event tracing capabilities.

## 3. System Model and Problem Statement

Penetration targets healthcare organization networks because they manage enormous volumes of sensitive patient data. Healthcare networks are still susceptible to changing cyber threats, even with various security mechanisms in place. It takes more than just conventional embedding technology to incorporate image data security, which is complicated. When considered, poor performance and lower payload capacity were achieved.

**Figure 1:**
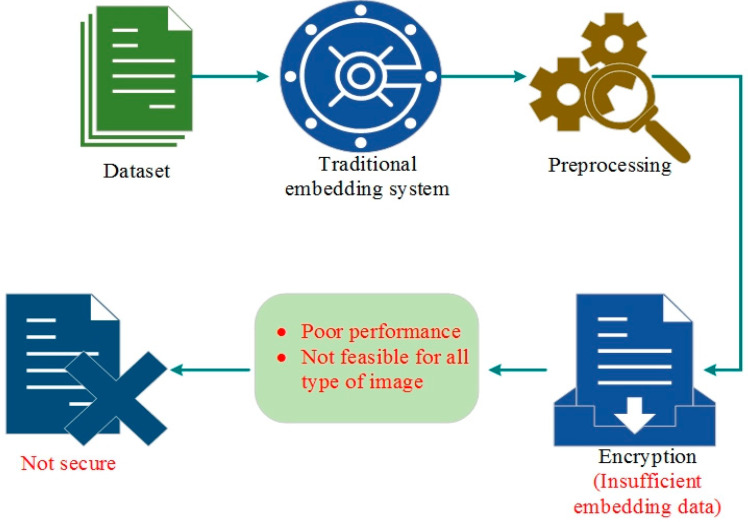
Difficulties with the traditional method.

Usually, the embedding scheme is utilized for the digital application to secure the image data. But in many cases, the embedded algorithms are not sufficient to hide the data because of poor quality images and a lack of security features in embedding algorithms. Hence, it is not suitable for all image data and does not provide the expected best outcome. So, it is not secure against the malicious event, which is defined in Figure 1.

## 4. Proposed GCbTS to Secure the Healthcare Network Data

**Figure 2:**
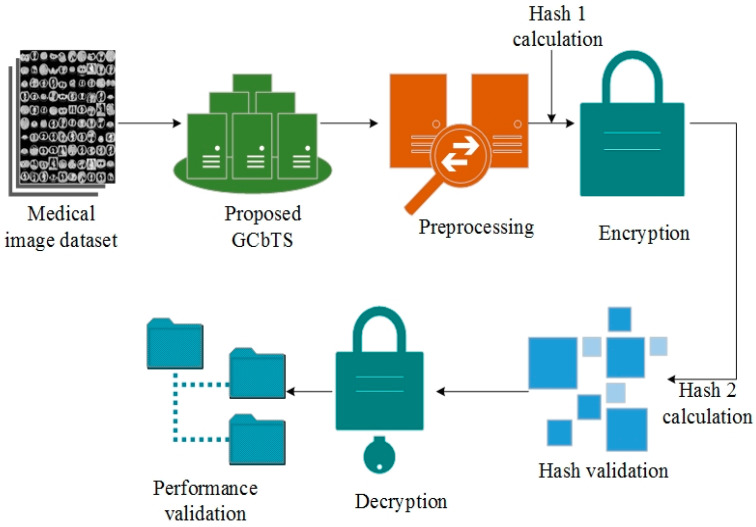
Proposed architecture.

This study introduces a new framework called the Graph Convolution-based Twofish Security (GCbTS) Framework to enhance the security of medical images in healthcare networks. The proposed framework is constructed utilizing suitable parameters and is subjected to testing using the collected data. The medical photos obtained from the Kaggle site were collected to evaluate the developed system. The structure of the suggested GCbTS approach is depicted in Figure 2 and Algorithm 1.
**Algorithm 1 Graph Convolutional-Based TwoFish Security (GCbTS)**  
***Start***
  
***{***
   
*int* 
$I(D)=\{M_{1},M_{2},......M_{n}\}$
   
*//initialization of input digital video dataset*
  
***Preprocessing()***
  ***{***
    
*int P,*
δ
*,*
x
*,*
y
    
*//Preprocessing variables are initialized.*
    P(D)→δ(x−y)   
*//Noises are removed.*
   
***Hash 1 calculation ()***
   
***{***
   
*int* 
$H_{v1}$
*,*
p
   
hash_1→Mod(D,p)
   
*// The hash 1 value is estimated and stored in the framework.*
   
image→frame
   
*//Input videos are split into the types of frames.*
   
***}***
   
***Encryption()***
   
***{***
   
*Int* 
$E_{i}^{*}$
*,*
n
*,*
$E^{*}$   
$E_{i}^{*}\to E^{*}(M_{1},M_{2},.....M_{n})$
   
*//By twofish algorithm, the images are encrypted and assembled.*
    
***}***
    
***Hash 2 calculation()***
    
***{***
    
$hash\_2\to Mod(E_{n}^{*},p)$
   
*// The hash 1 value is computed for encrypted data.*
  
***}***
  
***Hash validation()***
  
***{***
   
*if* 
$\left(H_{v1}=H_{v2}\right)$
   
***{***
 
*//Verification is successful, and the system sends encrypted data* 
*along with key.*
***}***  
***}***
  
***Decryption()***
   
***{***
$N_{d}^{*}\to E_{i}^{*}\;/S_{k}$*//Encrypted data are retrieved into their original form.* 
***}***
  
***}***
  
***Stop***



The first step involves gathering the medical image dataset and calculating the hash 1s. After collecting the data, preprocess them to reduce the noise. The created key is then used to encrypt these preprocessed images throughout the encryption process. To confirm the user’s identity, hash 2 is also calculated for the encrypted data and compared with hash 1. Users can access the data in their original forms after they have been verified and encrypted using 156 the shared key.

### 4.1. Process of GCbTS Model

**Figure 3:**
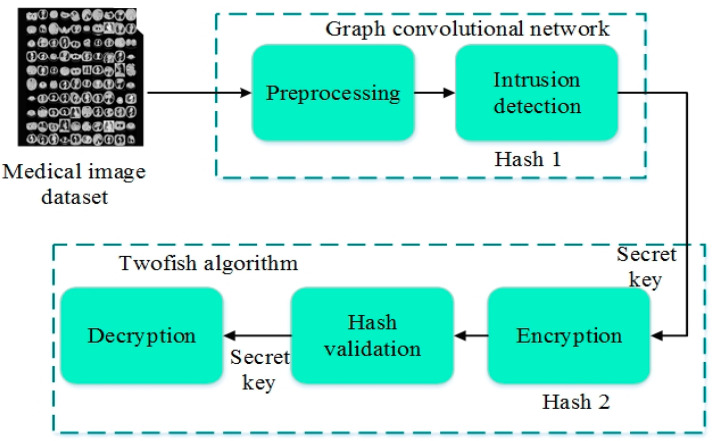
The process of the GCbTS.

The security framework comprises a graph convolutional neural network [26] and the twofish algorithm [27]. The medical image data are initially collected and included in the suggested framework for validation. The proposed approach is a graph-based convolutional neural network comprising the input layer, two concealed layers, and an output layer. The intended network’s design is shown in Figure 3.

The input layer incorporates the collected medical photos. At hidden layer 1, the hash calculation takes place after preprocessing the medical images. The neural network sequentially identifies and eliminates the assault images in the input image. Moreover, the twofish model handled the crypto mechanism. The proposed methodology is suitable for all distributed data environments by the graph convolutional neural network. To accept and process all formats of the image data type, the graph convolutional neural network was executed. It contains the three layers, which are the input layer, the dense layer, and the output layer. In the dense layer, the twofish encryption process was executed.

In addition, considering other encryption models, the twofish algorithm was designed and tested against different crypto analysis. So, it is more secure than other encryption models, and for this reason, the twofish algorithm is adopted for this research study. For this reason, the present proposed network has received a high confidential score.

#### 4.1.1. Data Initialization and Preprocessing

The dataset has been initialized in the framework using graph convolutional neural features. Equation (1) describes the data initialization procedure:

(1) $$I(D)=\{M_{1},M_{2},........M_{n}\},$$

where *I*(*D*) represent the data initialization function, *D* represents the dataset, *M* represents the input medical image data, and *n* represents the images collected from the healthcare network. The noise in the images extracted from the dataset complicates the ongoing process. Thus, the preprocessing stage is done to remove any noise from the dataset. Furthermore, the complexity has been lowered with the use of function preprocessing. As a result, the computing time is shortened, producing high results. Equation (2) removes the noise features from the input dataset:

(2) P(D)=S+δ(x,y)×[x−y].

Here, *P*(*D*) represents the data preprocessing variable, *δ* denotes unwanted noise tracking parameters, *x* represents input data features, and y represents noise features present in the data. Thus, the preprocessing process removes the noise features.

Following this preprocessing function, the structure computed and stored the hash value for the input images. Equation (3) calculates the hash value. The hash 1 value is calculated by concatenating the randomly chosen prime number with the binary data on the input image [28]:

(3) $$H_{v1}=S\;\;Mod\;p,$$

where *H*_*v*1_ represents the initial image hash 1 value, and *p* is denoted as a prime variable number. Then, the supplied datasets are entered into the encryption stage with their calculated hash values. The subsequent part provides an elaborate elucidation of the forthcoming procedures.

#### 4.1.2. Encryption

Decoding and encryption are the two main steps in crypto analysis. In this case, the data are encrypted using the twofish algorithm. The initial generation of the key encrypts the data. In this case, the bit widths of the input data block’s controlling variables are used to generate the key. The technique then uses the generated key to encrypt the images. Equation (4) provides the encryption procedure:

(4) $$E_{i}^{*}=E^{*}(\sum_{i=1}^{I}Ms),$$

where *E*^∗^ denotes the encrypted image, *i* represents the total images, and *E*^∗^ represents the encrypting factor. Meanwhile, the encryption process changes the input medical images into a different format. Here, the image is considered as the data; primarily, the binary values of the images were extracted based on the most significant bit and least significant bit [29]. Then, for the binary data, an encryption process is performed to hide the information, as described in Equation (4). From this encrypted data, the following hash 2 was evaluated.

#### 4.1.3. Hash Verification

Following the encryption of the data, two hash values are calculated using the encrypted data. The user is comparing the value of hash 1 with the projected hash 2 for the encrypted data. The determined hash values should correspond to the specified hash 1 value for the original material. The calculation of hash 2 is denoted by Equation (5):

(5) $$H_{v2}=E_{s}^{*}(Mod\;\;p).$$

Here, *H*_*v*2_ denotes the 2nd hash function, and *E*^∗^ represents the encoded dataset. To guarantee user confirmation, either hashing 2 and password 2 values are validated when the hash algorithm 2 computations are finished. Equation (6) confirms it.

(6) $$Verify=\left\{\begin{array}{c} if(H_{v1}\neq H_{v2})\;\;\;\;Not\;verified\\ if(H_{v1}=H_{v2})\;\;\;\;user\;verified \end{array}\right..$$

The system only gives access to the user when the sharing key matches the hash values. If this does not happen, the system will deny the user access. Here, hash 1 is valued by the owner, who encrypts the message, and hash 2 is measured by the receiver, who needs to decrypt the message. Hence, if hash 1 and hash 2 are equal then the data are not injected, and in this way, they are used for verification.

#### 4.1.4. Decryption

Decryption is the act of deciphering encrypted data. Upon completing the user validation process, the computer system will provide the user with the authentication code. Once the system confirms the user’s authenticity, it decodes the encrypted videos to retrieve the original footage. This procedure is referred to as the decryption process. Equation (7) describes the decryption procedure:

(7) $$N_{d}^{*}=E_{i}^{*}/S_{k}=D.$$

Here, *N*^∗^ denotes the decrypted data, *D* represents the initial original data, and *S*_*k*_ denotes the secret key. The initial picture format was recreated by merging the decrypted images after the encrypted data were cracked utilizing an encryption key during decryption.

## 5. Result and Discussion

Graph Convolutional-Based Twofish Security (GCbTS) is suggested to protect pictures on a medical network. The medical images are gathered and arranged into datasets, then added to the recommended architecture. After the data are initialized, the video undergoes preprocessing to remove noisy features. The hash value of each image is subsequently calculated and stored. The twoish algorithm then combines and encrypts the photos using the provided key. The system authenticates the user by using hash 1 to calculate and validate the hash 2 values. The software delivers the encrypted video and the decryption key if the consumer’s validation is successful. The execution variables are compiled in Table 1.

Furthermore, the results of the GCbTS architecture are evaluated. Moreover, an analysis of costs and benefits was done to assess the efficacy of the suggested approach.

### 5.1. Case Study

The unique GCbTS system is evaluated using the medical MNIST dataset downloaded from the official Kaggle website. The collection comprises a total of 58,954 pictures. This set of images, which is comparable to the MNIST dataset, is made up of 64 × 64 medical photos. The present-day arrangement of the photographs was created after they had been taken from different datasets.

**Figure 4:**
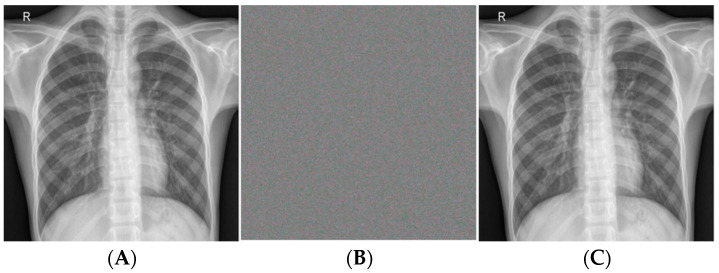
(**A**) input image, (**B**) encrypted image, and (**C**) decrypted image.

**Figure 5:**
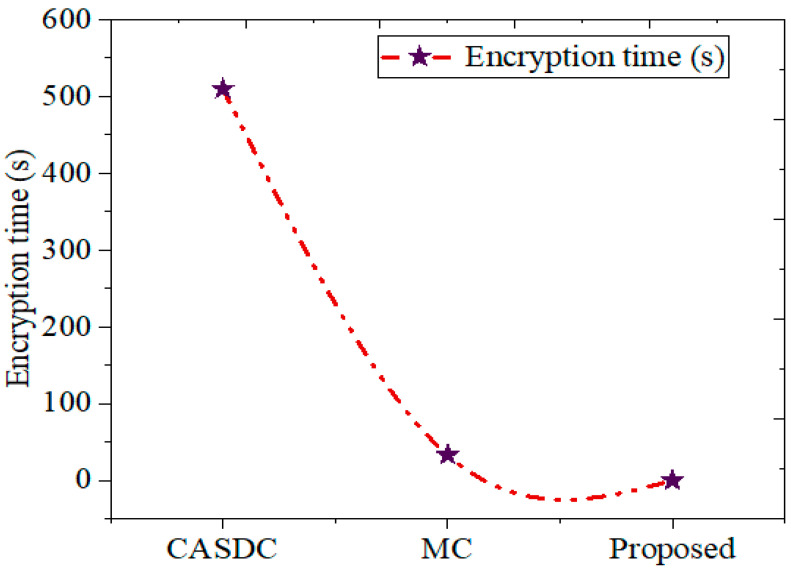
Encryption time comparison.

This case study aimed to scrutinize the system’s design process. The dataset first collects and incorporates the medical images into the recommended framework. Once the digital video datasets are gathered, each image is preprocessed to reduce noise and split into a certain number of images. The first step involves determining the hash value. The considered image data for this research investigation process and the encryption–decryption are defined in Figure 4 and in this case study section. Here, the decryption is only eligible after the successful verification of hash 1 and hash 2. Figure 5 intimates the encryption time comparison. Thus, the message transaction was secure with high integrity.

The previously produced key is then used to encrypt the preprocessed photos. To verify the user’s identity, hash 2 was computed and contrasted with hash 1. After verification, the shared key decrypts the data and returns them to their original condition so users can access them. To determine the degree of improvement, we assess the outcomes generated through the GCbTS paradigm by comparing these with the results from earlier techniques. Furthermore, Figure 5 presents the data acquired from the encryption and decryption procedures.

### 5.2. Performance Analysis

The Python-based GCbTS framework was created on a Windows 10 system and subsequently compared to other methods to demonstrate its superior performance. These include a cryptographically secure data system (CASDC) [30], message cryptography (MC) [29], a quantum-inspired cuckoo search algorithm (QICSA) [31], a quantum-inspired tabu search algorithm (QITSA) [31], and a quantum-inspired genetic algorithm (QIGA) [31].

#### 5.2.1. Encryption Time

“Encryption time” is the time needed to finish the encryption process for medical imaging data. It is commonly expressed in seconds. An algorithm is superior in efficiency if its encryption time is smaller.

The GCbTS framework’s encrypting time has been compared to other widely used methods to verify its encryption speed. It is compared to other known approaches in Figure 5. The CASDC and MC methods achieved encryption times of 509.66 s and 33.40 s, respectively. The reduced encryption and decryption time verified the seed of the proposed system to test this security process against the malicious events.

#### 5.2.2. Decryption Time

The decryption time refers to how long it takes to decode encrypted data back into their original image form. Cryptographic keys or algorithms reverse the data’s encryption in this operation.

**Figure 6:**
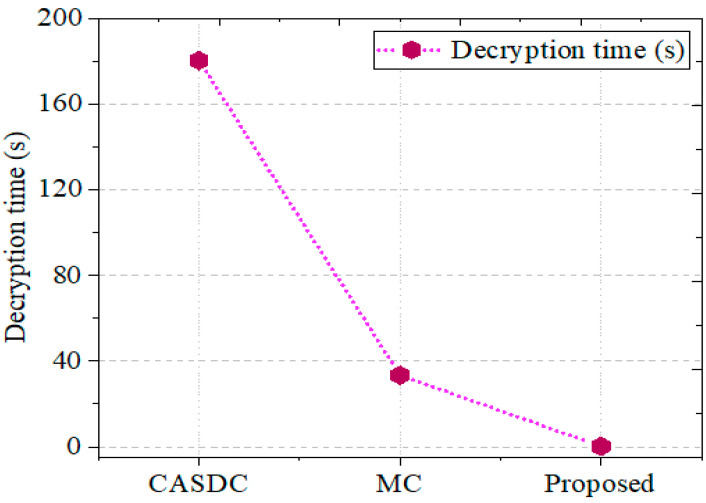
Decryption time comparison.

To validate the GCbTS framework’s decryption time, the decryption time is compared to other successful approaches. Figure 6 compares it to other known techniques. The CASDC and MC methods achieved decryption times of 1802 s and 33.40 s, respectively.

#### 5.2.3. MSE

The mean squared error is a widely used metric for quantifying the mean square variance between the actual and anticipated values in a repression problem. It is a way to measure how accurate the model’s forecasts are. Equation (8) expresses the formula for mean squared error (*MSE*):

(8) $$MSE=\frac{1}{d}\sum_{i=1}^{d}(x_{i}-{\hat{x}}_{i}{)}^{2},$$

where *d* denotes the number of taken data points, *x*_*i*_ denotes the actual values of points, and *i* denotes the compressed points. Gaining the lower *MSE* with other traditional models has revealed a better quality of image after the decryption process.

**Figure 7:**
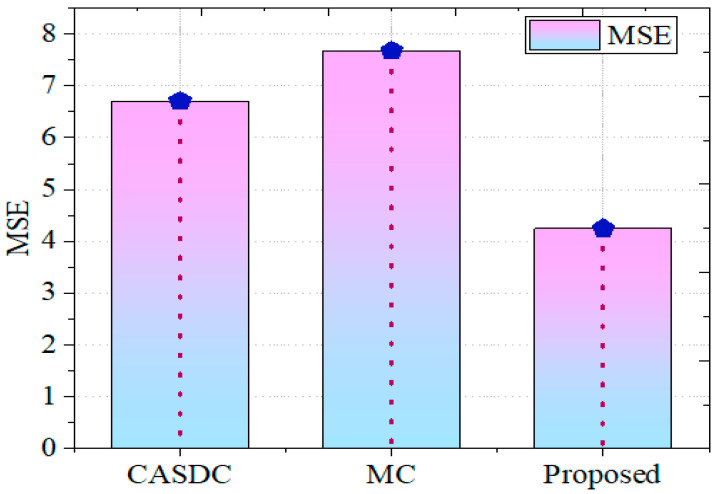
MSE comparison.

The GCbTS framework’s mean squared error (*MSE*) is evaluated against several effective techniques for MSE validation. In Figure 7, it is compared with other well-established methods. High mean squared errors (MSEs) of 6.7064 and 7.6702 were obtained by the CASDC and MC techniques, respectively.

#### 5.2.4. PSNR

People commonly utilize the peak signal-to-noise ratio (*PSNR*) metric when evaluating the accuracy of compressed or reconstructed images. *PSNR* is useful for assessing the caliber of cryptographic changes made to medical images when protecting healthcare networks from intrusion. Equation (9) provides the formula for calculating *PSNR*. Compared to the traditional models, the proposed model scores the highest PSNR as 24.7, which indicates that after the encryption and decryption process, the image quality was not degraded. Also, the proposed method is suitable for securing image data:

(9) $$PSNR=10.\log_{10}\left(\frac{255^{2}}{MSE}\right)$$

**Figure 8:**
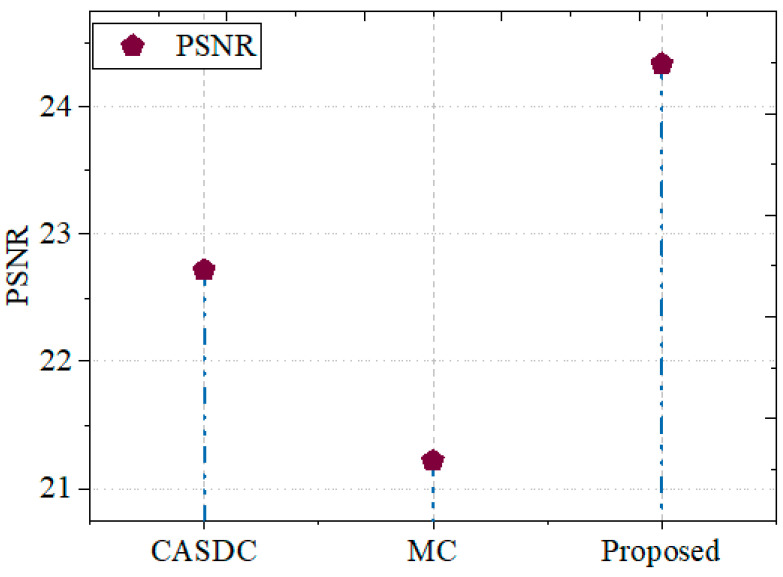
PSNR comparison.

When comparing the *PSNR* of the GCbTS framework to other successful methods, the PSNR for the GCbTS framework is compared. It is contrasted with other prevailing techniques in Figure 8. The CASDC and MC methods achieved PSNR values of 22.7171 and 21.2168, respectively.

#### 5.2.5. Throughput

Throughput refers to a network’s effective encryption, transmission, and decryption rate. It indicates the volume of data that a network can manage or transfer within a given time frame. The higher throughput defined that the present model is effective in the data transmission process.

**Figure 9:**
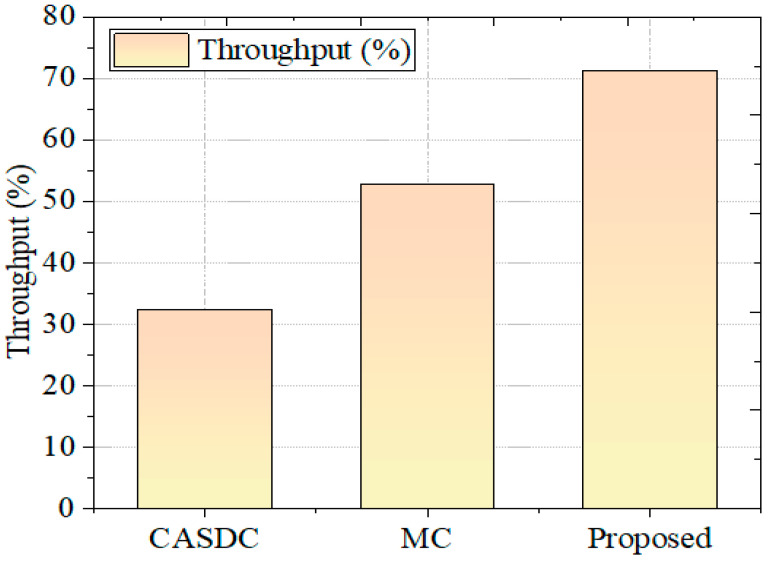
Throughput comparison.

The efficiency provided by the GCbTS framework is validated by contrasting it with the throughput of other effective techniques. Figure 9 compares it to other known approaches. The CASDC and MC methods achieved 32.37% and 52.79% throughput rates, respectively.

The numerical results for all comparison data are given in Table 2. Here, all the traditional models were tested in the same proposed platform, which afforded an accurate outcome and made the comparative study more accurate. For the security proof, the confidentiality score is measured for all methods in Table 2.

#### 5.2.6. Error Rate

The error may be used to describe inconsistencies or errors that arise during cryptographic procedures. Various factors, including data corruption, incorrect encryption or decryption techniques, and deliberate attacks, may cause these problems.

**Figure 10:**
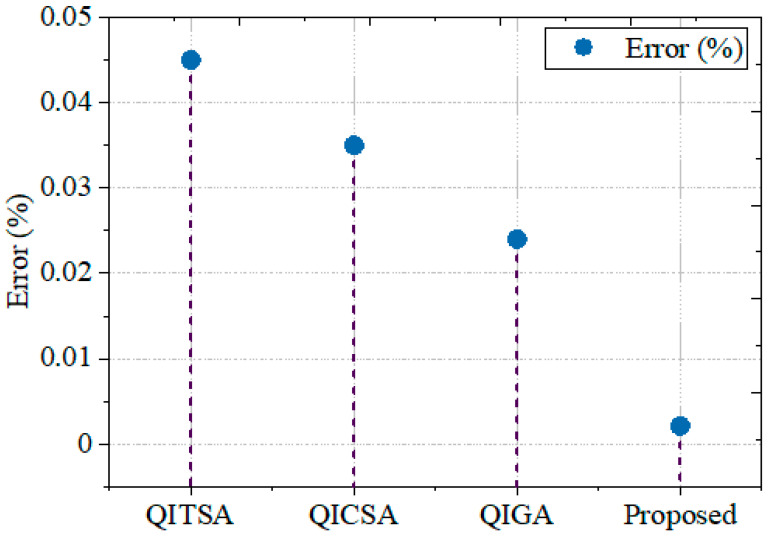
Error rate comparison.

The error rate of the framework is compared with other well-known techniques to validate the GCbTS framework’s decrypting time. In Figure 10, it is compared with other widely used methods. Earlier techniques, such as QITSA, QICSA, and QIGA, attained error rates of 0.045%, 0.035%, and 0.024%, respectively. The overall proposed outcome is tabulated in Table 3. The proposed research is executed for the distributed environment, and it is not tested for quantum computers and quantum algorithms. To do the post-quantum cryptography in the present work, the database has to be converted to quantum bits, and then the post-quantum crypto algorithm needs to be implemented to secure the generated quantum bits.

## 6. Conclusions

Securing healthcare data is more important in digital smart work; hence, the present work introduced a novel GCbTS as a security mechanism to protect medical data against third parties. Here, the owner and the third parties were identified by performing the hash 1 and 2 verification. Furthermore, the proposed model achieved 24.721 PSNR, 71.225% throughput, and a reduced average error rate of 0.002%. In addition, the average cryptographic processing time for both encryption and decryption is 0.2 s. By verifying all those outcomes, the presented GCbTS has improved the security performance by 20%. Hence, the proposed model is more apt for this specific domain to secure the digital sensible data for healthcare and other applications. However, this current research work does not address the memory space requirement or the need for energy resources.

In the future, incorporating the memory computing model and energy needs estimation along with its optimization procedure will provide the most precise reliability outcome. The key demerit of this study is that it has not been tested with quantum attacks. In the future, introducing post-quantum algorithms and testing them with quantum attacks will produce a better outcome.

## Figures and Tables

**Table 1:** Implementation parameters.

Implementation Parameters	
Parameter	Description
Programming platform	Python
Version	3.7.14
Operating system	Windows 10
Dataset	Medical MNIST
Data format	Image
Dataset size	6 GB
Processing power	20 mW
Cryptographic algorithm	Twofish algorithm

**Table 2:** Overall comparison.

Method	Encryption Time	Decryption Time	Throughput	MSE	PSNR	Confidentiality Score (%)
CASDC	509.66 s	1802 s	3237.2	6.7064	22.7171	77%
MC	0.3340 s	0.3340 s	5279.7	7.6702	21.2168	72%
Proposed	0.225 s	0.231 s	71.225	4.245	24.721	98%

**Table 3:** Performance statistics of GCbTS.

Metrics	Performance
Encryption time	0.225 s
Decryption time	0.231 s
MSE	4.245
PSNR	24.721
Confidentiality score	98%
Throughput	71.225%
Error	0.0021%

## Data Availability

Kaggle.
